# Combined macromolecule biomaterials together with fluid shear stress promote the osteogenic differentiation capacity of equine adipose-derived mesenchymal stem cells

**DOI:** 10.1186/s13287-021-02146-7

**Published:** 2021-02-12

**Authors:** Mohamed I. Elashry, Nadine Baulig, Alena-Svenja Wagner, Michele C. Klymiuk, Benjamin Kruppke, Thomas Hanke, Sabine Wenisch, Stefan Arnhold

**Affiliations:** 1 Institute of Veterinary Anatomy, Histology and Embryology, Justus-Liebig-University of Giessen, Frankfurter Str. 98, 35392 Giessen, Germany; 2 Clinic of Small Animals, c/o Institute of Veterinary Anatomy, Histology and Embryology, Justus Liebig University of Giessen, 35392 Giessen, Germany; 3 Institute of Veterinary Physiology and Biochemistry, Justus Liebig University of Giessen, 35392 Giessen, Germany; 4 Institute of Materials Science, Max Bergmann Center of Biomaterials, Technische Universität Dresden, Budapester Str. 27, 01069 Dresden, Germany

**Keywords:** Stem cells, Osteogenic differentiation, Biomaterials, Fluid shear stress

## Abstract

**Background:**

Combination of mesenchymal stem cells (MSCs) and biomaterials is a rapidly growing approach in regenerative medicine particularly for chronic degenerative disorders including osteoarthritis and osteoporosis. The present study examined the effect of biomaterial scaffolds on equine adipose-derived MSC morphology, viability, adherence, migration, and osteogenic differentiation.

**Methods:**

MSCs were cultivated in conjunction with collagen CultiSpher-S Microcarrier (MC), nanocomposite xerogels B30 and combined B30 with strontium (B30Str) biomaterials in osteogenic differentiation medium either under static or mechanical fluid shear stress (FSS) culture conditions. The data were generated by histological means, live cell imaging, cell viability, adherence and migration assays, semi-quantification of alkaline phosphatase (ALP) activity, and quantification of the osteogenic markers runt-related transcription factor 2 (*Runx2*) and alkaline phosphatase *(ALP*) expression.

**Results:**

The data revealed that combined mechanical FSS with MC but not B30 enhanced MSC viability and promoted their migration. Combined osteogenic medium with MC, B30, and B30Str increased ALP activity compared to cultivation in basal medium. Osteogenic induction with MC, B30, and B30Str resulted in diffused matrix mineralization. The combined osteogenic induction with biomaterials under mechanical FSS increased Runx2 protein expression either in comparison to those cells cultivated in BM or those cells induced under static culture. *Runx2* and *ALP* expression was upregulated following combined osteogenic differentiation together with B30 and B30Str regardless of static or FSS culture.

**Conclusions:**

Taken together, the data revealed that FSS in conjunction with biomaterials promoted osteogenic differentiation of MSCs. This combination may be considered as a marked improvement for clinical applications to cure bone defects.

**Supplementary Information:**

The online version contains supplementary material available at 10.1186/s13287-021-02146-7.

## Introduction

Cell-based stem cell therapy together with biomaterials is a rapidly growing approach for tissue regeneration. The aim is to repair and restore normal tissue function by means of stem cells together with biological macromolecules as scaffold materials. Such approach may provide a new therapeutic option for the treatment of chronic degenerative disorders that are currently incurable with ordinary routine applications.

Osteoporosis is a disease causing bone fragility, risk of fracture, and insufficient bone healing due to deterioration of the bone microarchitecture [1, 2]. Scarcity of effective treatment has encouraged seeking for alternative therapeutic approaches [3–5]. Currently, autologous and allogenic bone grafts are the most appropriate approach to restore bone defects [6]. However, the limited bone availability and the necessity of an invasive and expensive surgical intervention have fostered the promotion of the development of substitute materials.

Using biomaterials substitutes for bone tissue engineering allows developing biological scaffolds capable of restoring, maintaining and improving tissue function [7]. Developing a biological substitute to refill bone defects requires a combination of a proper scaffold biomaterial that maintain a suitable niche for mesenchymal stem cell (MSCs) differentiation [8, 9]. Certain criteria have to be considered to select the biomaterial: (1) it should mimic the target tissue components in terms of biocompatibility and biodegradability, (2) it has biomechanical and osteoinductive properties, and (3) the biomaterial construct must be easy to establish, efficient, and economical to facilitate clinical application [6, 8]. A novel biomimetic silica-collagen hybrid nanocomposite has been developed that meet the required criteria [10]. Inspired by the siliceous basal spicules of marine glass sponges, it represents a combination of the organic bovine tropocollagen type I and inorganic silicate [11]. Both ingredients of this nanocomposite are physically found in bone tissue and have been proven to exert a positive influence on bone healing. Collagen is the most abundant framework protein in mammalian tissues which provides a promising component of a biomaterial due to low antigenicity, low inflammatory, and low cytotoxicity. Moreover, it has the ability to promote cell adhesion, proliferation, and differentiation and it is responsible for biocompatibility, controlled biodegradation, and the maintenance of signal transduction [11–13]. Silicon is one of the most abundant trace elements in living organisms. A group bunch of evidence demonstrated the important role of silicon for bone metabolism and formation that have been proven under in vitro and in vivo investigations [14, 15]. It has been found that silicon not only increased the collagen synthesis within the extracellular matrix of human osteoblasts but also it promoted their proliferation and differentiation [16–18]. In addition, it plays an important role as an initiator for bone mineralization and formation [19, 20]. Furthermore, silicification reinforces the collagen fibrils leading to improved mechanical stability of the biomaterial [21]. The combined silica and collagen provides inorganic/organic composite with promising characteristics regarding chemical, structural, and technological parameters for the biomaterial suitable for bone engineering [13, 21, 22]. Currently, a group of evidence has shown the effectiveness of collagen based microcarrier scaffold for MSC proliferation and chondrogenic differentiation [23]. A similar study has combined CultiSpher-S Microcarrier (MC) with chitosan hydrogel and revealed an enhanced metabolic activity and surface adhesion of chondrocytes suggesting the usefulness of the composite for tissue regeneration [24]. The criteria of cell viability, adhesion, and detachment would determine the quantity of cells available for regeneration and production of the therapeutic effect. Additionally, the porosity of MC and surface adhesion support cell attachment, nutrient transfer, and a reservoir system to deliver active biological molecules [25].

MSCs are multipotent undifferentiated adult stem cells, extractable from various tissues including bone marrow and adipose tissue. These cells have the ability for self-renewal and differentiation potential into bone, fat, and cartilage [26]. The osteogenic differentiation of MSCs provides a valuable tool for bone tissue engineering. Furthermore, the accessibility, straightforward isolation, and immunomodulatory properties of MSCs demonstrate their usefulness in cell-based therapy for bone regeneration [27]. However, the effect of biomaterials on the cell viability, adherence, migration, and osteogenic differentiation of MSCs are not fully elucidated. Thus, the present study aims to investigate the effect of biomaterial substitutes on equine adipose tissue-derived MSC morphology, viability, adherence, migration ability, and osteogenic differentiation potential. MSCs were co-cultivated in conjunction with collagen MC, nanocomposite xerogels B30 and B30 in conjunction with strontium (B30Str) biomaterials. These combinations were allowed to differentiate into the osteogenic fate up to day 21 either under static (ST) or mechanical fluid shear stress (FSS) culture conditions. Data were generated by histological investigations as well as by evaluation of cell viability, cell adherence, and the migratory potential. Osteogenic capacity was assessed by semi-quantification of the alizarin red S staining (ARS), alkaline phosphatase (ALP) activity, and quantification of the osteogenic markers runt-related transcription factor 2 (*Runx2*) and alkaline phosphatase (*ALP*) expression. Our data revealed the influence of various scaffolds biomaterials on stem cells criteria including morphology, attachment, and cell migration. We show enhanced cell viability in the presence of MC and mechanical FSS. An increased ALP activity in the osteogenic differentiation together with B30 under static culture suggests the usefulness for bone formation in vitro. We report that mechanical FSS together with MC improves matrix mineralization. We show that combined mechanical FSS together with MC, B30, and B30Str increases osteogenic differentiation of MSCs as shown by upregulated *ALP* and *Runx2* expression. All together underpinned the effect of various biomaterials on osteogenic commitment of MSCs which suggest their usefulness for regenerative applications in veterinary medicine.

## Materials and methods

### Preparation of the biomaterial scaffolds

#### Nanocomposite xerogels (B30 and B30Str)

The silica-collagen nanocomposites were kindly provided from the Max Bergmann Center of Biomaterials and Institute of Materials Science from Dresden University of Technology, and preparation was carried out as described by [11, 22]. Our investigations were performed with xerogel granules. Therefore, the silica-collagen monoliths were ground into a powder and then classified into different particle fractions. In these investigations, fractions with a particle size < 0.125 mm were examined.

#### CultiSpher-S microcarrier (MC)

Preparation of the CultiSpher macroporous gelatin microcarriers was performed according to the company instruction (Percell, Biolytica AB, Sweden). Briefly, the dry MC were rehydrated in phosphate buffered saline (PBS, 1 g/50 mL) for 1 h then were autoclaved at 121 °C for 15 min. The PBS was removed and fresh PBS was added then MC was washed three times in culture medium. Generally, 1 × 10^5^ cells were mixed with 0.1% MC in Dulbecco’s Modified Eagle’s Medium (DMEM) for all experimental setups.

### Isolation of adipose tissue-derived MSCs

Experiments were accomplished from equine adipose MSCs (*N* = 8; aged 7.66 ± 1.34 years). Equine MSCs of both sexes were examined as previously reported by [28]. Briefly, the adipose tissue was obtained from the subcutaneous fat from horses being slaughtered at the local abattoir and the Institute of Veterinary Pathology, Justus-Liebig University, in Giessen. MSCs were transferred in cold PBS supplemented with 1% penicillin-streptomycin (P/S, Gibco® life technologies, Germany). Adipose tissue samples were cut into 1 mm^2^ pieces using a sterile scalpel then were washed twice in PBS for 5 min. The samples were digested under shaking in 0.1% collagenase I (Biochrom AG, Germany) in 1% bovine serum albumin dissolved in PBS for 60 min at 37 °C. The homogenates were filtered in 70 μm cell strainer and were centrifuged at 240*g* for 5 min. The supernatant was discarded, and the pellets were suspended in fresh 1 g/L DMEM, Gibco® life technologies, Germany). The cells were counted using a hemocytometer and were expanded in DMEM supplemented with 10% fetal bovine serum (FBS, Biocell, Germany) and 1% P/S in T75 culture flasks. The cells were incubated at 37 °C, 5% CO_2_, and 90% humidity under a standard culture condition up to confluency. Upon confluency, cells were detached using TryplE (Gibco® life technologies), were counted using a hemocytometer, and were stored in 1 mL aliquots at − 160 °C. Medium change was carried out twice a week, and cells of passage 2–4 were used for the following experiments. MSCs used in the current study were examined for the positive stem cell markers including CD 90, CD 44, and CD 105 as well as the negative marker CD 45 using flow cytometry as previously reported from our group (Supplementary Fig. 1a) [29]. Additionally, the expression of CD 90, CD105, Nanog, and Oct 4 in MSCs were evaluated using polymerase chain reaction (PCR) as previously shown [30].

### Induction of shear stress using a rotating bioreactor

After expansion, 4 × 10^6^ cells and 20 mg of MC and B30 biomaterials were cultivated in 10 mL growth medium using two different settings: a static culture in falcon tubes (Sarstedt, Germany) and a rotating bioreactor (Rotary Cell Culture Systems, Synthecon Inc., Houston, TX, USA). To enable a proper gas exchange, the falcon lid was perforated before incubation. In the rotating culture, the vessel was allowed to perform 11 cycles/min. Both settings were incubated at 37 °C with 5% CO_2_ for 4 days without carrying out a medium change. Afterwards, the medium with biomaterial-cell-complexes (from both experimental settings) was carefully transferred to a new 15-mL falcon tube. Two washing steps followed by 5 min interval until all complexes were precipitated were done. The latter was suspended in 10 mL fresh medium for 5 min to select all non-attached cells using various sedimentation speeds. The harvested complexes were used for the consecutive experiments.

### Histological staining

The biomaterial-cell-complexes were fixed with 4% paraformaldehyde (PFA, Roth, Germany) for 20 min at 4 °C followed by two washing steps in distilled water. The cell biomaterials complexes were dehydrated in ethanol (Roth, Germany), were cleared in xylene (Roth, Germany), and were processed for paraffin embedding. Sections of 6 μm were produced using a microtome (Leica, Germany), were transferred to glass slides (Roth, Germany), and were processed for hematoxylin and eosin histological staining to get an overview of cell-biomaterial interaction. The sections were mounted using Eukitt mounting media (HICO-MIC, Hirtz & Co, Germany) and were examined using the light microscope equipped with a digital camera and the LAS V4.4 software (Leica, Germany).

### Phalloidin staining

Complexes were fixed in 4% PFA, were washed twice for 5 min interval, and were then incubated with phalloidin, an actin filament cytoskeleton staining (2, 5%, Sigma-Aldrich, Steinheim, Germany) for 30 min. The nuclei were counterstained with 0.05% Hoechst dye in TBS for 5 min (Invitrogen, USA). After staining, the complexes were transferred in PBS buffer on microscopic slides and were examined using the fluorescence microscope (Axio Observer.Z1, Carl Zeiss, Germany).

### Scanning electron microscopy

To examine the morphology of the biomaterial surface and the appearance of attached cells, a scanning electron microscopy (SEM) was performed. Cell-biomaterial complexes were fixed in 2% glutaraldehyde in cacodylate buffer (Merck, Germany). The specimens were dehydrated in ethanol gradient for 10 min and afterwards and were coated by hexamethyldisilazane (Merck) overnight. After drying, specimens were sputter-coated with Au/Pd with a Balzer sputter coater (SCD 020, Balzers Union, Germany) and were examined by a Digital Scanning Microscope (DSM 940, Zeiss at 15 kV and 16 mm working distance). The cell-biomaterial complexes were photographed using a Digital Image Scanning System 5 and a Digital Image Processing System 2.9 (Point Electronic, Germany).

### Transmission electron microscopy (TEM)

To assess the ultramicrocellular morphology of the cell-biomaterial in combined culture, the complexes were fixed in cacodylate buffer solution (pH 7.2) containing 2% PFA, 0.02% picric acid (Fluka, Germany) and glutaraldehyde (Sigma-Aldrich, Germany) for 24 h at 4 °C. After that, the complexes were post fixed in 1% osmium tetroxide (Sigma-Aldrich, Germany) in 0.1 M cacodylate buffer for 2 h at room temperature. The complexes were counterstained with 0.5% uranyl acetate (Delta Microscopy) for 30 min and 0.2% lead citrate for 80 s. Then, complexes were dehydrated and were embedded in Epon (Sigma-Aldrich). Ultrathin 70 nm sections were generated using an ultramicrotome (Reichert - Jung Ultracut, Leica Microsystems) and were examined using a TEM (EM 109, Zeiss, Germany).

### Live cell imaging

To investigate the cell viability and the capacity to spread out from the biomaterial after having attached on their surface, live cell imaging was carried out (Supplementary video 1, 2, and 3). Cell-biomaterial complexes were placed into a 35 × 10 mm culture dish (VWR, Germany) with 3 mL in growth medium and then incubated for 1 h followed by live cell imaging under standard culture conditions using Axio Observer Z1, Temp Module S, CO_2_ Module S (Zeiss, Germany). The images were taken every 15 min up to 72 h. The analysis was performed using the Axiovision Imaging Software (Zeiss, Germany). Afterward, the complexes were fixed in 4% PFA for 20 min and were stained in phalloidin.

### MTT cell viability assay

To evaluate the viability of cells already attached on the biomaterial, a colorimetric MTT assay (3-4,5-dimethylthiazol-2yl-2,5-diphenyltetrazolium bromide, Sigma-Aldrich, Steinheim, Germany) was performed. Viable cells are able to reduce the MTT yellow substrate to the insoluble blue formazan; the latter can be solubilized and measured by a spectrophotometer reader at a specific wavelength. Therefore, combined 2 × 10^6^ cells with MC, B30, and B30Str were cultivated in growth medium under standard culture conditions for 4 days. After washing twice in PBS, the cells and biomaterials were incubated with MTT for 3 h. The cells were lysed with 200 μL of dimethylsulfoxide (DMSO, AppliChem, Germany) together with a tissue Lyser in oscillation frequency of 50 HZ (Quiagen, Hilden, Germany) for 5 min. Hereafter, the color intensity was measured by a microplate reader at 570 nm (Tecan, Germany) and was analyzed with Magellan™ – Data Analysis Software (Crailsheim, Germany).

### Osteogenic differentiation induction

To examine the osteogenic differentiation capacity of combined cultivation of MSCs together with biomaterial (B30, B30Str, and MC), the cells were cultivated in growth medium for 48 h to facilitate cell attachment on biomaterials. The cells were allowed to differentiate in osteogenic medium containing DMEM, 5% FBS, 0.05 mM ascorbic acid-2-phosphate (Sigma-Aldrich, Germany), 10 mM β-glycerolphosphate (Sigma-Aldrich, Germany) and 0.1 μM dexamethasone (Sigma-Aldrich, Germany) up to 21 days. Cells were provided with fresh medium twice a week. Cells were incubated in parallel either in basal medium (BM, 5% FBS in DMEM and 1% P/S) or osteogenic differentiation (OD) medium without biomaterials were served as control (NC). Osteogenic differentiation was assessed by evaluating the morphological alteration, histological examination using ARS staining, alkaline phosphatase (ALP) activity, and osteogenic relative markers at days 7, 14, and 21 post induction.

### Mechanical fluid shear stress

MSCs were seeded in combination with MC, B30, and B30Str in a ratio 1 × 10^5^ per well in a 24-well culture plates (VWR, Germany). After 48 h, the growth medium was replaced by the OD medium. The plates were divided into two experimental groups: static (ST) and mechanical fluid shear stress (FSS) culture conditions. In the latter, the cells were incubated under a regular vertical rocking pattern (Supplementary Fig. 1b) [31]. To optimize the mechanical stress, a rocking angle of 10° with a frequency of 40 turns/min and a fluid depth of 3.13 mm were generated. The formula for the FSS calculation was previously described by [32]. The assumed fluid viscosity was 10^−3^ Pa s; the FSS was 77.21 mPa (in non-SI units 0.77 dyn/cm^2^) at the center of the dish bottom. The experimental setup guaranteed that the cells were covered with medium during cycling of the plates. In parallel, cells which were grown in parallel in BM served as negative control.

### Alizarin red S staining (ARS)

ARS-specific dye with affinity to calcium ions was used as an indicator for “Ca^2+^” deposition in the mineralized matrix. Fixed cells in 4% PFA were washed in distilled water three times at 5 min interval. The cells were incubated with 1% ARS staining solution (pH 4.2, Roth, Germany) at room temperature for 30 min. Excess of staining was washed three times in distilled water for 5 min to remove the unbound dye. The morphological alterations following each time point were examined and were photographed using a light microscope equipped with a digital camera and the LAS V4. 4 Software (Leica, Germany).

### Semi-quantification of ARS staining

ARS stained cells were washed twice in distilled water then were incubated with a volume of 2 mL of 10% cetylpyridinium chloride (CPC, Roth Germany) with a moderate shaking for 60 min. An equal volume from each experimental group was transferred to a 96-well microplate. The absorbance was measured in triplicates at 562 nm using microplate reader (Tecan, Germany).

### Measurement of alkaline phosphatase (ALP) activity

Cells were incubated with 500 μL of 1% Triton™ X-100 in 10 mM Tris (pH 7.4) at 4 °C for 60 min. The cell/biomaterial complexes were transferred into a 1.5 mL Eppendorf tube. Cell lysates were centrifuged at 28,400*g* for 2 min at room temperature and kept on ice. P-Nitrophenylphosphate (2 mg/mL) was dissolved into buffer solution containing 1 M Tris and 5 mM MgCl_2_ (pH 9.0). A volume of 50 μL of cell lysate was incubated with 150 μL of p-Nitrophenylphosphate solution for up to 30 min. The mixtures were loaded in triplicates into 96-well microplates. The standard of p-Nitrophenol solution was used to prepare the standard curve. ALP potency to hydrolyze p-Nitrophenylphosphate into p-Nitrophenol (PNP) was measured as previously reported [33]. The absorbance was measured at 405 nm by using a microplate reader. The rate of ALP activity was measured using the equation, *Y* = m**x** + b, where *x* = sample absorbance value and *y* = pNP concentration of samples.

### Real-time quantitative polymerase chain reaction (RT-qPCR)

Total RNA was extracted from cells co-cultivated with MC, B30, and B30Str at days 7, 14, and 21 post osteogenic induction using an innuPREP RNA mini kit (Analytik, Jena AG, Germany) and was quantified using a UV spectrophotometer (Bio-Photometer, Eppendorf AG). Cells were incubated in parallel either in BM or osteogenic medium without biomaterials and were served as controls (NC). Briefly, RNA samples were incubated with 3.9 μL per sample DNase mix containing 1.2 μL of 25 mM MgCl_2_, 1.2 μL DNase buffer I, 1.2 μL DNase I, and 0.3 μL RNase inhibitor in 100 μL PCR tubes. The mixture was incubated at 37 °C for 25 min then at 75 °C for 5 min in a thermal cycler (Bio-Rad, Germany). RNA samples were reverse transcribed into cDNA in a master mix containing 45 μL RT-Mix, 3 μL RNase inhibitor, and 3 μL reverse transcriptase multiscribe (Promega). The reaction was generated at 21 °C for 8 min, at 42 °C for 15 min, at 99 °C for 5 min, and at 5 °C for 5 min in the Bio-Rad thermal cycler. To examine the validity of the PCR reaction and the quality of the primers used, a qualitative PCR reaction was performed. All primers were purchased from microsynth, Germany, and were listed in Table 1. *Glyceraldehyde-3-phosphat dehydrogenase* (*GAPDH*) was used as an endogenous reference. PCR fragments were allowed to run in a 2% agarose gel electrophoresis at 180 V for 20 min and were examined under UV light. Relative osteogenic markers *Runx2* and *ALP* were quantified using RT-qPCR after 14 and 21 days. The reaction of 1 μL of cDNA template with 9 μL of a mixture composed of 5 μL 2 x SYBR Green PCR Master Mix (Bio-Rad), 0.6 μL of 10 pM forward and reverse primers mix, and 3.4 μL nucleic acid free water was carried out. The samples were loaded in triplicates in a 96-well PCR plates in a real time cycler (CFX96, Real-Time PCR Detection System, Bio-Rad). The following PCR condition was followed: activation at 95 °C for 2 min, 40 cycles at 95 °C for 15 s, and 60 °C for 30 s, followed by a melting curve (60–95 °C for 3 s). PCR-Mix without adding cDNA was served as a negative control. The qPCR data was relatively normalized to *GAPDH* expression. The expression of each target gene was analyzed using 2^−ΔΔCt^ equation as reported by [34].

**Table 1:** Primers sequences used for RT-qPCR analysis

Gene	Forward	Reverse	Size (bp)
GAPDH	CCAGCAAGAGAAGGAGAAAGG	AACTGTGGAGGTCAGGAGAC	93
Runx2	ACCGACAGCCCCAACTTC	ACCCGCCATGACAGTAACC	132
ALP	GACTGGTACTCGGACAACGAG	GTTCTTGGGGAACATGTACTTC	137

### Western blot analysis

To detect the protein levels of the selected osteogenic marker Runx2, a Western blotting experiment was performed. Cells from all experimental groups were lysed in a buffer consisting of 1 M urea, 2% sodium dodecyl sulfate (SDS), 10% glycerine, 0.01% bromophenol blue, and 6.25 mM Tris–HCl for 2 min. Cell lysates were collected, and protein concentrations were determined using the Bradford assay (Bio-Rad, Hercules, CA, USA). The protein samples were reduced using 5% 2-mercaptoethanol (Sigma-Aldrich, Germany), and 50 μg of protein samples was loaded into 7.5% SDS-polyacrylamide gel electrophoresis (PAGE). The separated proteins were transferred onto nitrocellulose membranes (Pall Bio Trace) at 350 mA for 90 min in a blotting chamber (VWR). Membranes were blocked with PBST (PBS, 0.1% Tween) containing 5% skimmed milk powder at 4 °C. The membranes were washed twice in PBST for 5 min and then were incubated with anti Runx2 mouse monoclonal primary antibody (1:500 diluted in PBST, sc-390,715, Santa Cruz) for 1 h at room temperature. Protein samples were processed in parallel using a mouse monoclonal anti β actin (1:10 diluted in PBST, DSHB) and were used as an endogenous reference. The membranes were washed 3 times in PBST at 5 min interval and then were incubated with goat-anti-mouse IgG (Dianova) horseradish-peroxidase-conjugated secondary antibody (1:5000) for 45 min at room temperature. The membranes were washed 5 times in PBST at 5 min interval. The protein bands were developed using ECL Select Western Blotting Detection Reagent (GE Healthcare, RPN2235) with *Amersham Hyperfilm* (#28906836).

### Statistical analysis

The influence of combined osteogenic induction together with MC, B30, and B30Str biomaterials on the osteogenic differentiation of MSCs was investigated. Data were collected under ST and FSS conditions at days 7, 14, and 21 post induction. In order to analyze the effect of biomaterials (MC/SC, MC/RC, B30/ST and B30/RC) on cell viability compared to BM with no added biomaterials, one-way ANOVA was carried out. To evaluate the effect of biomaterials (MC vs. B30) at different time points (T0 up to 72 h) on the migration capacity of MSCs, a two-way ANOVA was examined. To assess whether various biomaterials alter the migration potential of MSCs, a two-tailed Pearson (*r*) correlation coefficient analysis was carried out. For the effect of osteogenic induction (OD vs. BM) concurrently with biomaterials (MC, B30, and B30Str vs. NC) on cell viability, ALP activity, ARS staining, and the osteogenic-related markers (*Runx2* and *ALP)* expression, a two-way ANOVA was carried out. Multiple comparisons were tested using Tukey’s and Sidak’s post hoc tests. The data from triplicates presented as the mean ± SEM, and *p* value ≤ 0.05 was considered to be significant. All the statistical analyses were carried out using Graph Pad Prism 7.0 (La Jolla, Canada).

## Results

### Morphological evaluation of MSCs cultivated with biomaterials

**Fig. 1:**
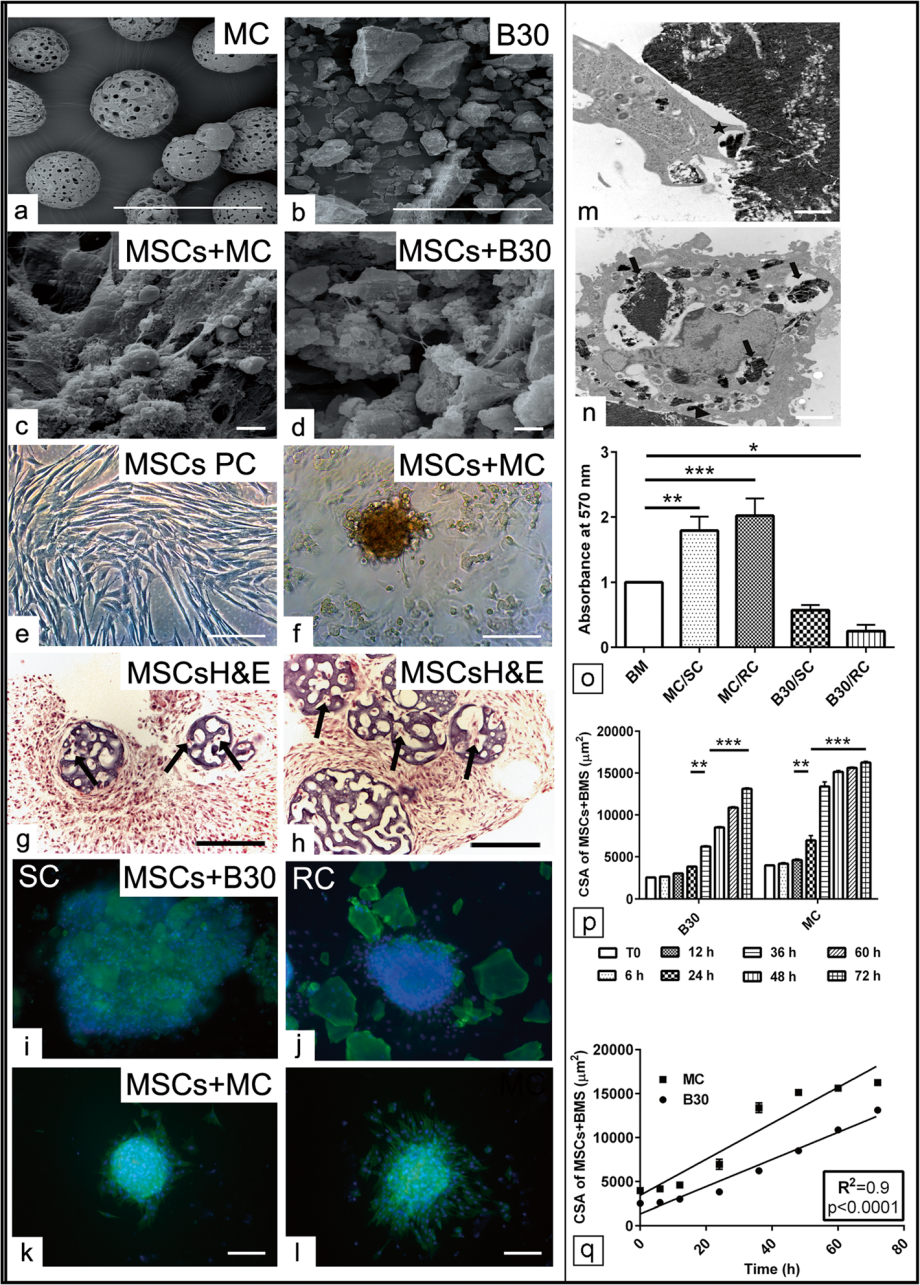
Morphological evaluation of MSCs cultivated with biomaterials. **a**–**d** Scanning electron microscopy (SEM) of equine adipose-derived mesenchymal stem cells (*N* = 8) cultivated in growth medium in conjunction with MC and B30 biomaterials for 48 h. **a**, **b** SEM of native CultiSpher-S MC and B30 biomaterials without MSCs. **c**, **d** SEM of combined cultivation of MSCs together with MC and B30 show cell adhesion, aggregation, and intercellular contacts. **e**, **f** Phase contrast (PC) images of MSCs seeded in a monolayer or in combination with MC. **g**, **h** Histological paraffin section of the MSCs pellet in conjunction with MC stained with H&E shows cell migration (black arrow) within the pores of the MC. **i**–**l** Combined cultivation of MSCs in conjunction with B30 (**i**, **j**) and MC (**k**, **l**) after static (SC) and mechanical rotation (RT) culture for 72 h show the pattern of cell adhesion and detachment from the pellet using phalloidin staining (green). Hoechst counterstain visualizes cell nuclei (blue). **m** TEM of combined MSCs and B30 biomaterial section show the phagocytic properties of MSCs to the biomaterials using podocytes extension (star). **n** TEM of MSCs section demonstrates various sized of B30 granules within the cell cytoplasm (black arrow). **o** Combined cultivation of MSCs with MC and B30 biomaterials under both SC and mechanical rotation culture (RC). Average quantification of cell viability using a microplate reader at 570 nm. Enhanced cell viability in the presence of MC compared to B30 can be shown. Cells cultivated in basal medium (BM) without any biomaterials were used as a negative control. **p** Average CSA measurement (μm^2^) for combined MSCs and biomaterial pellet up to 72 h (h, *N* = 4). **q** Linear correlation between the cultivation time point and the size of the MSCs and biomaterials (BMS, *N* = 4). All data presented as mean ± SEM. *p < 0.05, **p < 0.01, ***p < 0.001. Scale bar in **a**, **b** = 200 nm, **c**, **d** = 2000 nm, **e**, **f** = 100 μm, **g**, **h** = 70 μm, **i**–**l** =20 μm, and **m**, **n** = 2500 nm

In order to examine the morphology and the adherence capacities of MSCs cultivation in combination with biomaterials, MSCs were seeded in growth medium with MC and B30 and B30Str biomaterials. The SEM of the MC and B30 granules biomaterials without MSCs could be shown in Fig. 1a, b. Equine MSCs exhibited a complete coat around the MC (Fig. 1c). However, B30 granules of different shapes and sizes were combined by a plethora of MSCs forming large cell-granule-complexes. The xerogel granules themselves have a rather smooth surface with partially rough areas most likely formed by silica particles in conjunction with collagen fibrils. MSCs displayed cytoplasmic extensions for contacting adjacent cells and to facilitate secure attachment on the scaffold’s surface. Also, a solid network consisting of cells and cell cytoplasmic extensions with B30/B30str granules can be figured (Fig. 1c, d). In contrast, the typical spindle-like fibroblast appearance of MSCs was detected in the culture dish without adding the biomaterials (Fig. 1e); however, the cells seeded with MC formed a sphere-like accumulation and surface adhesion around these particles (Fig. 1f). The ordinary histological sections showed MSCs invasion through the pores of MC biomaterials indicating the cell viability (Fig. 1g, h).

In order to examine MSCs’ capacity to spread out of the biomaterials, two culture protocols were deployed. Combined MSCs together with B30 or with MC were cultivated either under static culture (SC) or mechanical rotation (RC) culture conditions. The complex was transferred into a culture dish and then a live cell imaging was recorded for 72 h. After static culture, the combined cultivation of MSCs and MC showed a homogeneous cells and biomaterial aggregates; however, combined MSCs and B30 displayed scattered biomaterial fragments with attached cells (Fig. 1i, j). Following the complex attachment to the culture dish, cells cultivated with MC under RC showed a comparatively a faster cell spreading out of the pellet compared to either MC under SC or B30 culture conditions (Fig. 1i–l). An abundant settlement of cells could be demonstrated either on the surface at single locations or in clusters for B30 and MC (Fig. 1i–l).

### Effect of biomaterials on MSC viability and migration potential

To get further insight into cell-scaffold interaction, cell motility was assessed following 4 days of SC and RC conditions using live cell imaging. Motility of cells that had attached to the different scaffold materials was studied at 15 min intervals over 72 h using the live cell imaging set up. Following the establishment of biomaterial/cell-cell attachment, cells started to grow out of the biomaterials and to spread in a radial fashion into the vicinity of the scaffolds via developing long cytoplasmic processes. In addition, those cells cultivated in conjunction with MC demonstrated enhanced viability under RC in comparison to SC condition (Fig. 1k, l). Interestingly, within the cytoplasm of stem cells migrating away from B30 granules, small particles can be detected. The quantity of internalized particles per cell is variable. Given the fact that B30/B30Str granules show a strong autofluorescence using the appropriate wave length suitable for the detection of the phalloidin staining, the assumption suggests that the cells were indeed able to incorporate parts of the B30 granules via autophagy in order to gradually degrading them. This observation was confirmed by TEM of the cells and pellet complexes (Fig. 1m, n). In some cases, after having migrated away from the scaffold material, MSCs were observed to re-invade the scaffold particles.

In order to examine the effect of combined cultivation of various biomaterials on MSC viability, MTT assay under SC and RC was carried out. After 5 days in growth medium, significant increases in cell viability for the cells cultivated with MC under both SC and RC condition (*p* < 0.01, *p* < 0.001) compared to the BM without biomaterial could be observed. In contrast, combined mechanical rotation with B30 displayed a reduction in the cell viability (*p* < 0.05) when compared to BM (Fig. 1o). To assess our data, a quantification of the cross sectional area (CSA) of the biomaterial with attached MSCs was performed at different time point following live cell imaging. The analysis revealed a significant increase in the CSA of B30/MSCs complex after 36 h (*p* < 0.01) compared to time 0 (T0). Then, a gradual increase of the size of the B30/MSCs complex was found up to 72 h (*p* < 0.001) compared to 36 h. In contrast, the cell migration out of the MC showed a different pattern considering the same time scale. The combined MSCs/MC complex demonstrated a faster increase in the CSA already after 24 h (*p* < 0.01) compared to T0. Interestingly, the CSA of the MSCs/MC complex showed a significant increase at 36 h (*p* < 0.01) compared to 24 h which remains stable up to 72 h (Fig. 1p). There is no significant alteration in the CSA of the pellet for both B30 and MC in the initial 12 h of the complex recording. Further data analysis revealed a significant linear correlation (*p* < 0.0001) between the migration speed of MSCs out of the pellet and the cultivation time (Fig. 1q). These data point out the pattern of cell migration out of the various biomaterials in a temporo-spatial comparison.

### Effect of biomaterials on the osteogenic differentiation of MSCs under static culture

**Fig. 2:**
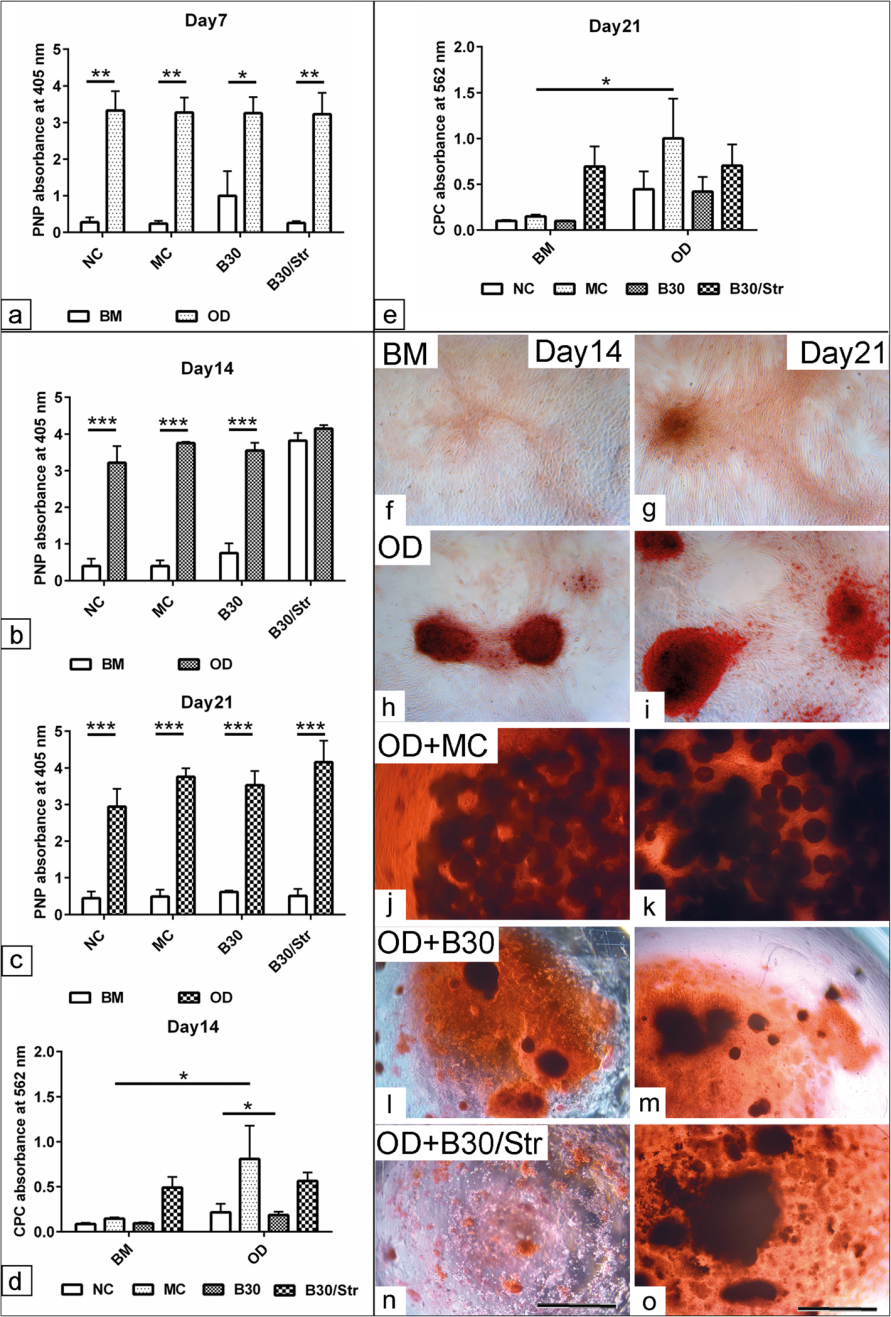
Effect of biomaterials on the osteogenic differentiation of MSCs under static culture*.* MSCs were cultivated in the presence of MC, B30, and B30Str biomaterials under standard culture condition for 48 h (*N* = 8 per experimental group). Cells were induced to osteogenic differentiation (OD) using 5% FBS in DMEM, 0.05 mM ascorbic acid-2-phosphate, 10 mM β-glycerolphosphate, and 0.1 μM dexamethasone up to 21 days. **a**–**c** Spectrophotometric quantification of alkaline phosphatase (ALP) activity at days 7, 14, and 21 shows promoted ALP activity post osteogenic induction. ALP activity was detected by measuring the metabolism of p-Nitrophenylphosphate into p-Nitrophenol (PNP) in the presence of ALP. The values of PNP were measured in triplicates at 405 nm absorbance, and the average was calculated. MSCs were grown in basal medium (BM) composed of 5% FBS in DMEM served as a negative control. Cells which were cultivated in osteogenic medium without adding biomaterials were used as negative control (NC). **d**, **e** Semi-quantification of the solubilized bound alizarin red S (ARS) using 10% cetylpyridinium chloride (CPC, Roth Germany) at days 14 and 21 post osteogenic induction (*N* = 8 per experimental group). The absorbance was measured in triplicates at 562 nm. Analysis of CPC revealed improved matrix mineralization as shown with MC biomaterial scaffold. **f**–**o** Representative ARS stained images (red) for MSCs cultivated in basal medium (BM, **f**, **g**), osteogenic differentiation medium (OD, **h**, **i**), OD + MC (**j**, **k**), OD + B30 (**l**, **m**), and OD + B30Str (**n**, **o**) under static conditions at day 14 and day 21. Osteogenic nodules could be shown in the presence of OD medium compared to BM. Diffused ARS staining was observed in the presence of OD + MC and scattered mineralization was observed in the condition of OD + B30 and OD + B30/Str. All data presented as mean ± SEM. *p < 0.05, **p < 0.01, ***p < 0.001. Scale bar in **f**–**o** = 100 μm

To understand the effect of various biomaterial compounds on MSCs fate, the cells were allowed to differentiate into the osteogenic lineage using a standard osteogenic induction cocktail under static culture condition. Evaluation of the alkaline phosphatase activity (ALP) as an indicator for osteogenic differentiation (OD) was performed up to 21 days post induction. The quantification of ALP activity at day 7 showed a significant increase in the osteogenic induced cells in conjunction with either MC (*p* < 0.01), B30 (*p* < 0.05), and B30Str (*p* < 0.01) or without adding biomaterials (NC, *p* < 0.01) compared to BM (Fig. 2a). Similar observations were found in all cell-biomaterial complexes at day 21 (*p* < 0.001) compared to the respective non-induced cells in BM (Fig. 2c). The MSCs/B30Str complex showed no significant change in ALP activity at day 14 compared to the control cells in BM (Fig. 2b). To assess the osteogenic potential using the different biomaterials, ARS staining was semi-quantified at days 14 and 21 using the CPC assay. The analysis of CPC values showed significantly increased values in the presence of MC together with osteogenic induction medium at day 14 (*p* < 0.05) compared to those cells cultivated only in BM. Furthermore, the combined osteogenic induction together with MC revealed increased CPC values (*p* < 0.05) compared to either osteogenic induction without biomaterials (NC) or the osteogenic induced cells cultivated with the B30 biomaterial (Fig. 2d). Similarly, an increased CPC absorbance could be found in cells which were cultivated in the presence of MC and the osteogenic differentiation medium at day 21 (*p* < 0.05) compared to the respective MSCs/MC complexes cultivated in the BM medium only (Fig. 2e). Although there was a moderate increase in CPC values for the cells cultivated with B30 or B30Str in the osteogenic medium detectable, the effect was not a statistically significant when compared with the respective biomaterials in BM. In order to confirm our data, a morphological examination of the osteogenic induced cells in combination with various biomaterials was carried out using ARS staining. Cellular aggregation-like nodules stained in red were detected using the standard osteogenic differentiation medium compared to BM conditions (Fig. 2f–i). By using combined MSCs with MC, B30 and B30Str revealed a diffused ARS staining at day 14 which became more disseminated at day 21 post induction (Fig. 2j–o).

### Effect of biomaterials on the osteogenic differentiation of MSCs under FSS culture

**Fig. 3:**
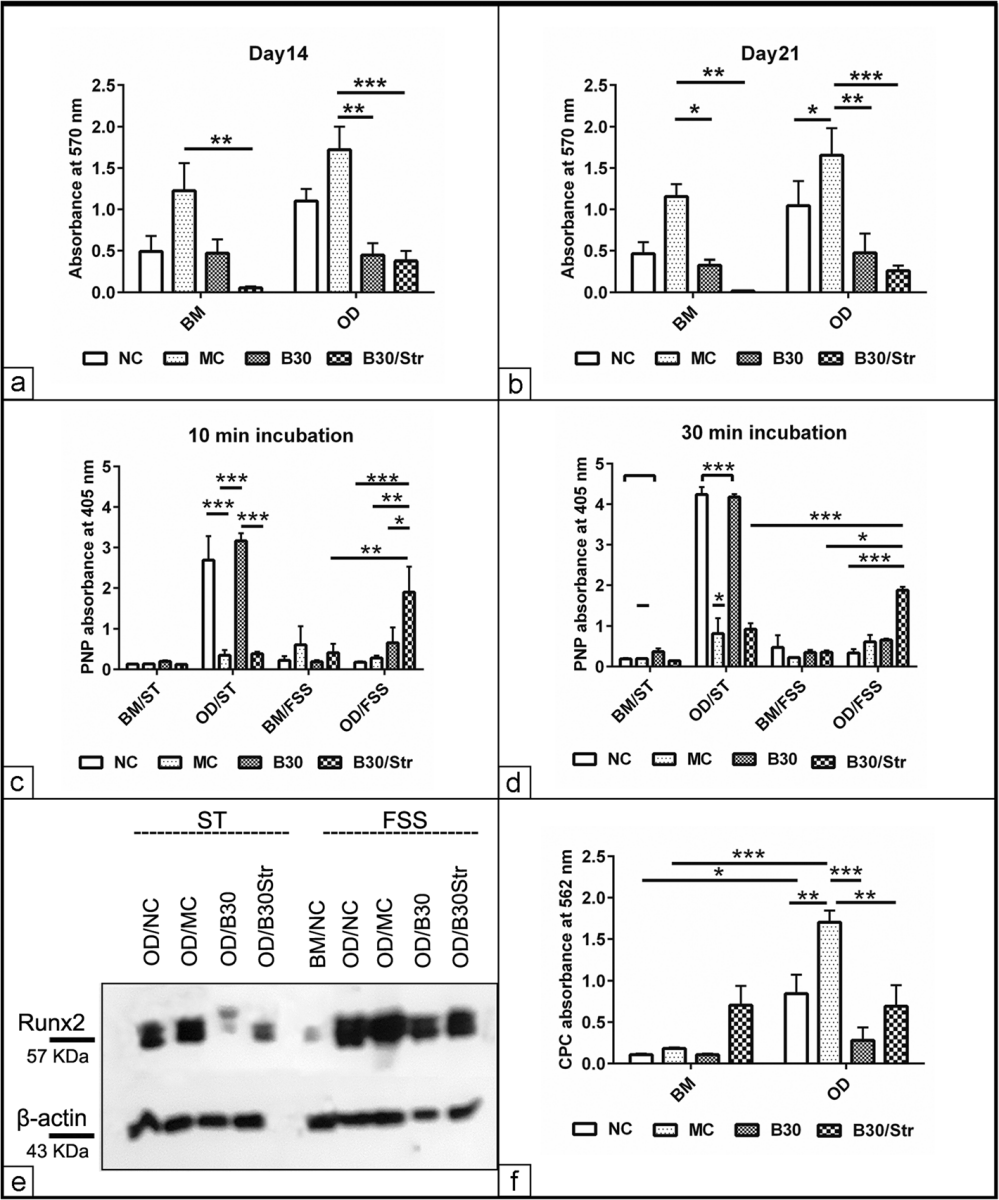
Effect of biomaterials on the osteogenic differentiation of MSCs under FSS culture. MSCs were grown in conjunction with MC, B30, and B30Str biomaterials under standard culture condition for 48 h to establish adhesion (*N* = 8 per experimental group). The cells/biomaterial complex was induced to the osteogenic differentiation (OD) fate under mechanical FSS in comparison to static (ST) culture up to 21 days. In parallel, MSCs which were grown in basal medium BM were served as negative control. Cells which were cultivated in osteogenic medium without adding biomaterials were used as negative control (NC). **a**, **b** Average cell viability was measured in triplicates at day 14 and day 21 using MTT assay. The data show enhanced cell viability in the presence of OD together with MC compared to B30, B30Str, and BM condition. **c**, **d** Spectrophotometric measurement of ALP activity in triplicates at 405 nm following 10 min and 30 min incubation. **e** Runx2 protein western blot following combined osteogenic differentiation (OD) induction with biomaterials (NC/OD, OD/MC, OD/B30, and OD/B30Str) under static (ST) and fluid shear stress (FSS) conditions at day 14. Approximately 50 μg of cell lysates were loaded in 7.5% SDS-PAGE in a reduced condition. Protein bands were transferred into a nitrocellulose membrane and were probed with anti Runx2 and β actin antibodies. Cells which were grown in basal medium (BM/NC) were used as negative controls. **f** Semi-quantification of the solubilized bound alizarin red S (ARS) in triplicates using 10% CPC at day 21 post osteogenic induction. Analysis of CPC (*N* = 8 per experimental group) revealed improved matrix mineralization as shown with MC biomaterial and control osteogenic condition without biomaterial (NC). All data presented as mean ± SEM. *p < 0.05, **p < 0.01, ***p < 0.001

In order to examine whether mechanical FSS influences the osteogenic differentiation of MSCs using various biomaterials, osteogenic induction under FSS culture condition up to day 21 was performed. The analysis showed that FSS clearly increased MSC viability in the presence of MC (*p* < 0.01 and *p* < 0.001) compared to B30 and B30Str following cultivation in BM. Furthermore, osteogenic induction in the presence of MC biomaterials enhanced cell viability (*p* < 0.01) compared with the combination of B30 and B30Str (*p* < 0.01 and *p* < 0.001) and the osteogenic differentiation medium at day 14 under FSS (Fig. 3a). Similarly, the combined FSS with MC biomaterials increased the viability of MSCs in comparison to the osteogenic induction without biomaterials (NC), with B30 (*p* < 0.01) and B30Str (*p* < 0.001) at day 21 regardless of the cultivation medium (Fig. 3b). Next, we evaluated whether FSS enhances ALP activity at day 14 using two incubation setups. After 10 min incubation, the analysis showed that osteogenic induction using osteogenic medium without biomaterials or in conjunction with B30 promoted ALP activity (*p* < 0.001) in comparison to MC and B30Str under static condition. Furthermore, combined osteogenic differentiation together with B30Str under FSS was able to increase ALP activity (*p* < 0.01) compared to the respective biomaterial in BM. In addition, FSS together with osteogenic differentiation in the presence of B30Str enhanced ALP activity compared to NC (*p* < 0.001), MC (*p* < 0.01), and B30 (*p* < 0.05) under the same condition (Fig. 3c). The same trend was observed after 30 min incubation in terms of increased ALP activity under combined osteogenic induction with MC (*p* < 0.05) compared to BM under static condition (Fig. 3d). The superior ALP activity was measured in the presence of B30 and the standard osteogenic medium (*p* < 0.001) compared to BM under static culture. Interestingly, however, osteogenic induction under FSS in the presence of B30Str increased ALP activity (*p* < 0.001) when compared with the same combination under static culture (Fig. 3d). To examine the effect of combined biomaterials and osteogenic medium on specific osteogenic marker at protein level, Runx2 was evaluated after 14 days following cultivation under ST and FSS conditions using western blot analysis. The data revealed that encoded Runx2 protein was detected at 57 KDa in a higher level in the combined osteogenic differentiation (SD) with MC compared to osteogenic differentiation without biomaterials, with B30 and B30Str under static culture. The combined osteogenic differentiation together with mechanical FSS increased the encoded Runx2 protein in all experimental groups either in comparison to those cells cultivated in BM or those cells induced under static culture (Fig. 3e). To assess matrix mineralization potential of various biomaterials under FSS, ARS was quantified at day 21 using the CPC absorbance assay. By comparing to the non-induced cells, osteogenic induction using MC biomaterial increased “Ca^2+^” deposition (*p* < 0.001). The combined FSS and osteogenic medium including MC biomaterials increased “Ca^2+^” deposition not only compared to standard protocol without biomaterials (NC, *p* < 0.01) but also in comparison to cultivation protocols involving B30 (*p* < 0.001) and B30Str (*p* < 0.01, Fig. 3f).

### Evaluation of the osteogenic differentiation marker expression

**Fig. 4:**
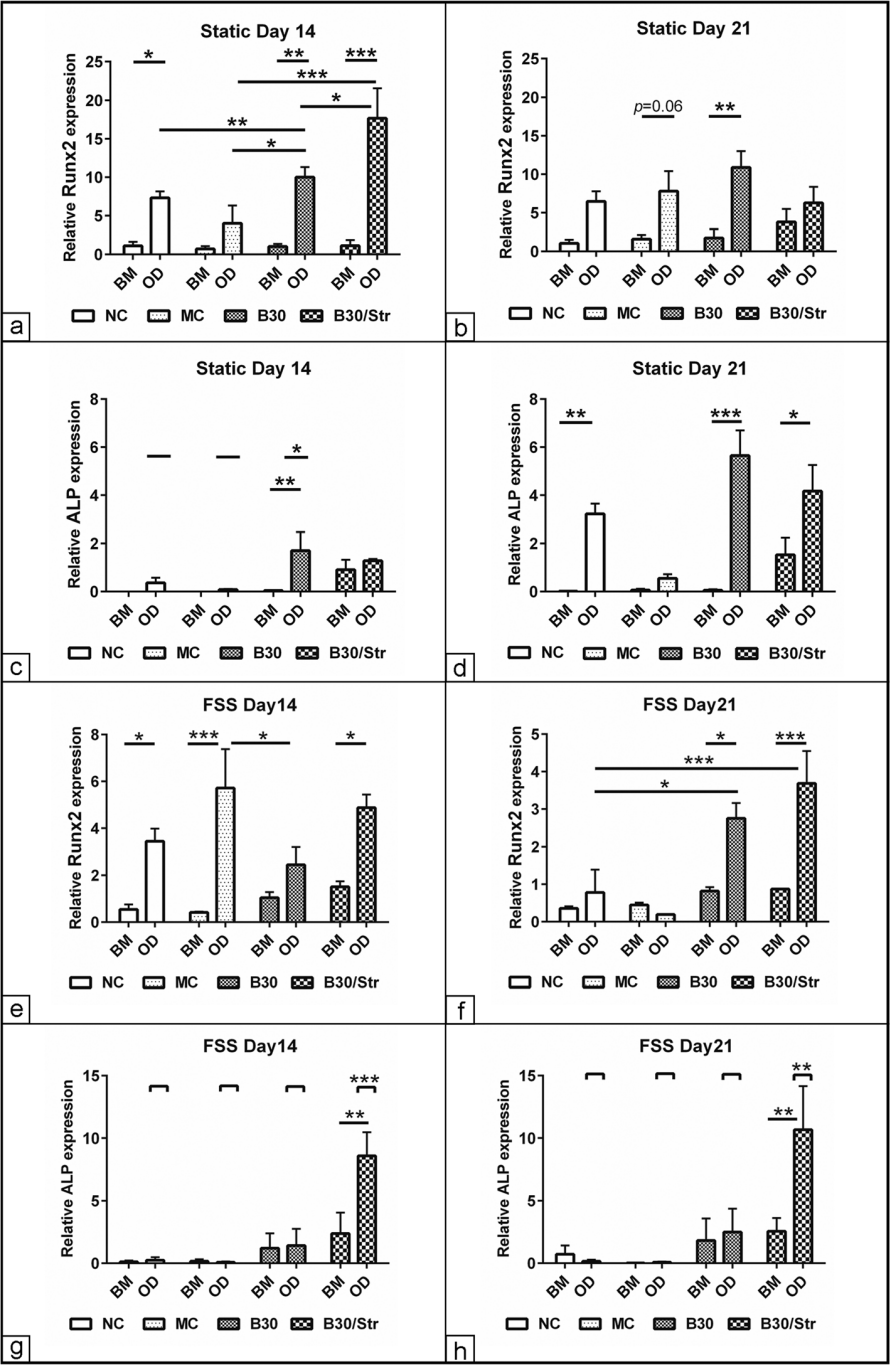
Evaluation of the osteogenic markers expression. MSCs were cultivated in osteogenic differentiation (OD) medium in the presence of MC, B30, and B30Str biomaterials under static and mechanical FSS culture condition (*N* = 8 per experimental group). Cells were osteogenic induced without adding biomaterials served as control (NC). Cells grown in parallel in basal medium (BM) were used as non-induced control. The levels of *Runx2* and alkaline phosphatase expression at day 14 and day 21 were quantified using RT-qPCR. **a** Relative *Runx2* expression at day 14 under static culture condition. **b** Relative *Runx2* expression at day 21 under static culture condition. **c** Relative *ALP* expression at day 14 under static culture condition. **d** Relative *ALP* expression at day 21 under static culture condition. **e** Relative Runx2 expression at day 14 under FSS condition. **f** Relative Runx2 expression at day 21 under FSS culture condition. **g** Relative *ALP* expression at day 14 under FSS culture condition. **h** Relative *ALP* expression at day 21 under FSS culture condition. The expression was normalized to non-induced cells in BM using 2^−ΔΔCt^ method [34]. *GAPDH* was used as an endogenous reference. The data presented as mean ± SEM. *p < 0.05, **p < 0.01, ***p < 0.001

In order to evaluate the effect of combined biomaterials together with osteogenic medium on MSCs differentiation, osteogenic relative marker expression was analyzed at days 14 and 21 under the static and FSS experimental setup. Osteogenic induction under static culture upregulated *Runx2* expression in the condition including osteogenic medium without biomaterials (NC, *p* < 0.05), B30 (*p* < 0.01), and B30Str (*p* < 0.001) compared to the respective control in BM at day 14. It was clearly shown that B30Str enhanced *Runx2* expression compared to NC, MC, and B30 experimental condition. *Runx2* expression was not detected under combined osteogenic induction and MC biomaterials (Fig. 4a). By using only B30 biomaterial, a significant upregulation of *Runx2* at day 21 could be achieved. At this later time point, combined MC and osteogenic induction showed a tendency of *Runx2* upregulation (*p* = 0.06) compared to the control cells in BM (Fig. 4b). Expression of *ALP* was only detected in the combined osteogenic induction together with B30 (*p* < 0.01) compared to matched cells in BM at day14. A similar upregulation of *ALP* expression using the combined osteogenic induction together with the B30 biomaterial was observed (*p* < 0.05) in comparison to osteogenic induction with MC or without cultivation with any biomaterials added at the same time point (Fig. 4c). Interestingly, however, *ALP* upregulation at day 21 was found in the presence of control osteogenic induction without any biomaterials (*p* < 0.01), B30 (*p* < 0.001), and in cells cultivated in the presence of B30Str (*p* < 0.05) in comparison to matched controls in BM. The expression of *ALP* was not detected in the protocol using combined MC and osteogenic medium at both day 14 and day 21 (Fig. 4d). Under mechanical FSS, the data revealed upregulated *Runx2* expression in three out of four of the experimental groups including osteogenic induction together with MC (*p* < 0.001), B30Str (*p* < 0.05), and control osteogenic without biomaterial (*p* < 0.05) compared to control in BM at day 14 (Fig. 4e). However, at day 21 of FSS stimulation, the level of *Runx2* was only detected in cells stimulated with osteogenic induction medium together with B30 (*p* < 0.05) and B30Str (*p* < 0.001) in comparison to the control in BM. Comparing the osteogenic induction using various biomaterials, *Runx2* expression displayed two-threefold increase in the B30 and B03Str-based osteogenic induction in comparison to either osteogenic differentiation alone or with MC biomaterials (Fig. 4f). The effect of FSS on *ALP* expression was also monitored at days 14 and 21. The data showed that *ALP* expression was only upregulated under combined osteogenic differentiation together with B30Str at both time points either compared to BM (*p* < 0.01) or compared to other experimental protocols (*p* < 0.001) (Fig. 4g, h).

## Discussion

Mesenchymal stem cells and biological bone substitutes offer a promising approach to restore bone defects. Such combination requires a proper selection of scaffold biomaterial that maintains a suitable niche for mesenchymal stem cell proliferation and differentiation. The ideal scaffold should provide a suitable vehicle for cell viability, adhesion, migration, proliferation, and differentiation. Optimization of such criteria would impact on the selection of the superior combination for regenerative medicine. In the current study, the effect of biomaterial bone substitutes on equine adipose-derived mesenchymal stem cell morphology, viability, adherence, migration, and osteogenic differentiation capacity were investigated.

The data revealed that scaffolds including MC, B30, and B30Str demonstrate suitable delivery vehicles for MSCs in terms of cell adhesion, viable networking, and migration within the pores of the biomaterials as shown by histological staining. In addition, MC-based biomaterial promoted cell viability compared to B30 and B30Str under both static and mechanical FSS. These data suggest that the construction of various biomaterials is a determining factor for MSCs growth and therapeutic potency. It has been found that combined cultivation of chondrocytes with microcarriers in a 3D fashion facilitates phenotype maintenance by delivering mechanical stimulation [35]. Furthermore, MC has the ability to support cell viability and cell adhesion [36]. Although biomaterials play a role for cell survival and proliferation [37, 38], it is questionable whether they also support MSCs differentiation. There are some negative examples. A study has shown that despite the silicon rubber microcarrier promoted cell growth and attachment, the functional differentiation of gastric stem cells was abolished [39].

In the present study, we have observed that MSCs detach from the biomaterial and migrate in a radial pattern. The data suggest enhanced cell viability since incompatible biomaterials could induce impaired cell viability and cytotoxicity. In some instances, cells internalize biomaterial particles into their cytoplasm during the detachment process as could be shown by TEM and phalloidin staining. These data are important criteria indicating the biocompatibility and biodegradability of the scaffold material under investigation. It has been documented that combined cell culture with collagenous microcarriers are favorable for three important aspects including cell attachment, cell proliferation, and cell detachment [40, 41]. In order to evaluate the cell migration out of the biomaterial, we quantified the CSA of MSCs and biomaterials complexes by using live cell imaging set up. The analysis revealed a faster cell detachment from MC compared to the B30 biomaterial in an observation period up to 72 h. The increases in the CSA of the cell pellet-MC complex together with enhanced cell viability and cell migration in to the pores suggest improved both cell viability and cell migration. These results could be referred to the morphological differences and basic construction of MC compared to B30. In the first biomaterial, the spherical form and the micropores provide stable cell adhesion conditions so that cells spread on the whole surface of MC. Thus, there is a large number of cells that may detach after the adhesion of the MC/cell conglomerate on the culture plate. In contrast, sharp granular surface of the B30 biomaterial with acute angles and absence of pores might lead to an unstable attachment and an impaired cell detachment. These data are congruent with the enhanced cell viability on MC. In agreement with our hypothesis, it has been documented that MC should provide a suitable cell attachment to tolerate mechanical stress but also not to impair cell detachment at the end of the regenerative process as previously reviewed [42]. Thus, our data point out the suitable support of cell migration out of the biomaterial, which should be the basis for the differentiation fate and an efficient tissue regeneration.

Our data revealed no significant alteration in the CSA of the combined cell pellet together with both B30 and MC in the first 12 h of the cultivation period. This aspect might refer to the time required to establish a proper cell niche with a stable and secure cell adhesion on the surface of the appropriate biomaterials before the induction of further cell networking.

The data of the presented study revealed that the ALP activity at day 7 and day 21 is increased by the osteogenic induction under static culture condition in all experimental setups with biomaterials. The osteogenic commitment was indicated by ARS semi-quantification particularly in the presence of combined MC and osteogenic medium. These data are in the same line with previous report that showed a rapid osteogenic differentiation, matrix mineralization, and upregulation of *Runx2* and *ALP* expression in periodontal stromal stem cells after cultivated with alginate-based MC [43]. The osteoinductive effect of MC could deliver stable cell adhesion via the gelatin coat and induction of matrix mineralization. In a similar study comparing the effect of enzymatic harvested and MC-bound cells regarding the osteogenic differentiation of human fetal MSCs, there was an upregulation of the osteogenic markers as well as matrix mineralization of MC bound cells [42]. Additionally, our data point out that there might be a link between the osteogenic commitment of cells and the structure of MC. It has been reported that gelatin-coated surfaces or MC fully generated from collagen enhance the osteogenic differentiation and matrix mineralization of human bone marrow MSCs as indicated by enhanced ALP activity, ARS staining, and upregulated *ALP* and *bone sialoprotein* expression [44]. On the other hand, the analysis of the osteogenic specific markers have shown that under static culture condition in the presence of an osteogenic induction medium, B30Str induced an early upregulation of *Runx2* and *ALP* expression at day 14. However, MSCs cultivated in combination with B30 biomaterial showed a later upregulation of *Runx2* at day 21. These data highlight the potential of biomaterials on the osteogenic induction of MSCs in a time-wise comparison. In agreement with our data, an earlier study has shown that calcium phosphate-based hydroxyapatite and a composite of degradable gel (BONITmatrix) have promoted the osteogenic differentiation of human bone marrow-derived MSCs even without the addition of any osteogenic induction medium as could be shown by an enhanced ALP activity and an upregulation of osteogenic markers [45]. The authors of this study concluded that the surface interaction of MSCs with a certain and suitable biomaterial already acts as an osteogenic stimulant even in the absence of an osteogenic induction medium. Furthermore, by integrating strontium within the B30 biomaterial, the osteogenic commitment was even promoted as shown by the upregulation of *Runx2* and *ALP* expression. In the same line, several reports revealed that strontium enhances the osteogenic differentiation and matrix “Ca^2+^” deposition which can be a useful tool for bone repair [46]. Another study from the same group reported about an enhancement of the osteogenic potential of human MSCs by a collagen-strontium-based hydroxyapatite scaffold. In the latter material, the included strontium activates bone formation via the β-catenin signaling pathway [47].

The data revealed that the combination of B30Str and the osteogenic medium enhanced the ALP activity indicating the osteogenic commitment. This could even be enhanced under mechanical FSS. These results indicate a double osteoinductive effect in which the applied FSS might mimic the in vivo bone formation under mechanical loading and the additional presence of strontium promotes “Ca^2+^” deposition in the vicinity of cells. These findings confirm reports suggest that strontium promotes osteoblasts viability and reduces the osteoclasts activity as previously reviewed [48]. Moreover, strontium has shown to promote the matrix mineralization by increasing the “Ca^2+^” level in the extracellular microenvironment which modulates the activity of “Ca^2+^” sensory receptor [49].

Surprisingly, the expression of *Runx2* and *ALP* was only weak under conditions of the osteogenic induction using MC under static culture conditions. However, the combined osteogenic medium with MC under FSS increased “Ca^2+^” deposition as shown by CPC analysis not only compared to standard protocol without biomaterials but also in comparison to a B30 and B30Str-based protocols.

The data clearly indicate that FSS enhances the osteogenic capacity of MC compared to static culture condition. This is also underlined by the increased *Runx2* expression under the stimulation of FSS in most experimental setups which was independent of the biomaterial used. In this context, previous studies by our and other groups have shown that the ectopic stimulation using “Ca^2+^” [28] and/or mechanical FSS [31] promoted osteogenic differentiation of MSCs. In the same line, the level of the encoded Runx2 protein was increased in the combined osteogenic differentiation with biomaterials under mechanical FSS compared to static culture condition as shown by western blot analysis indicating the osteogenic commitment.

## Conclusion

The present study investigates the impact of various biomaterials on the osteogenic differentiation potential of equine adipose tissue MSCs under static and FSS culture conditions. We provide evidence that especially the collagenous MC promoted cell viability and migration as well as matrix mineralization suggesting the usefulness of MC as a scaffold for cell-based therapy. All biomaterial constructs promoted ALP activity in the presence of osteogenic medium already under static non-stimulated culture conditions. We found that FSS together with MC increases cell viability which might be an important factor to enhance tissue regeneration. The data revealed that mechanical FSS enhanced osteogenic differentiation of MSCs as shown by increased ALP activity, upregulated *Runx2* and *ALP* osteogenic markers expression, and encoded Runx2 protein expression which could be a tool to promote bone regeneration. The data together revealed that FSS in conjunction with biomaterials improved osteogenic differentiation of MSCs that may be considered as a preliminary approach for clinical applications to cure bone defects.

## Supplementary Information

**Additional file 1: Supplementary Fig. 1a.** Flow cytometry analysis for specific stem cells markers including CD44, CD90, CD105, CD45 and major histocompatibility complex II (MHCII) for ASCs isolated from retroperitoneal (RP), subcutaneous (SC) and lipoma (LP) as previously reported from our group (Arnhold et al. 2019): *Investigation of stemness and multipotency of equine adipose-derived mesenchymal stem cells (ASCs) from different fat sources in comparison with lipoma. In Stem cell research & therapy 10 (1), p. 309.* **Supplementary Fig. 1b.** Calculation of the fluid shear stress (FSS) according to Zhou et al. (2010)

**Additional file 2.**

**Additional file 3.**

**Additional file 4.**

## Data Availability

The data collected and the analysis performed to generate the manuscript results are available from the corresponding author on reasonable request.

## Abbreviations

MSCs: Adipose-derived mesenchymal stem cells
MC: Collagen CultiSpher-S Microcarrier
B30: Nanocomposite xerogels B30
B30Str: Combined nanocomposite xerogels B30 with strontium
FSS: Fluid shear stress
ST: Static culture
OD: Osteogenic differentiation
BM: Basal medium
ALP: Alkaline phosphatase
Runx2: Runt-related transcription factor 2
GAPDH: Glyceraldehyde-3-phosphat dehydrogenase
ARS: Alizarin red S
H&E: Hematoxylin and eosin
