# Supplementary material for: Combined macromolecule biomaterials together with fluid shear stress promote the osteogenic differentiation capacity of equine adipose-derived mesenchymal stem cells
Source: Stem Cell Res Ther. 2021 Feb 12;12:116. doi: 10.1186/s13287-021-02146-7 (PMC7879632; doi:10.1186/s13287-021-02146-7)
Supplement: Supplementary file 1 — Additional file 1: Supplementary Fig. 1a. Flow cytometry analysis for specific stem cells markers including CD44, CD90, CD105, CD45 and major histocompatibility complex II (MHCII) for ASCs isolated from retroperitoneal (RP), subcutaneous (SC) and lipoma (LP) as previously reported from our group (Arnhold et al. 2019): Investigation of stemness and multipotency of equine adipose-derived mesenchymal stem cells (ASCs) from different fat sources in comparison with lipoma. In Stem cell research & therapy 10 (1), p. 309. Supplementary Fig. 1b. Calculation of the fluid shear stress (FSS) according to Zhou et al. (2010) [file 13287_2021_2146_MOESM1_ESM.docx]

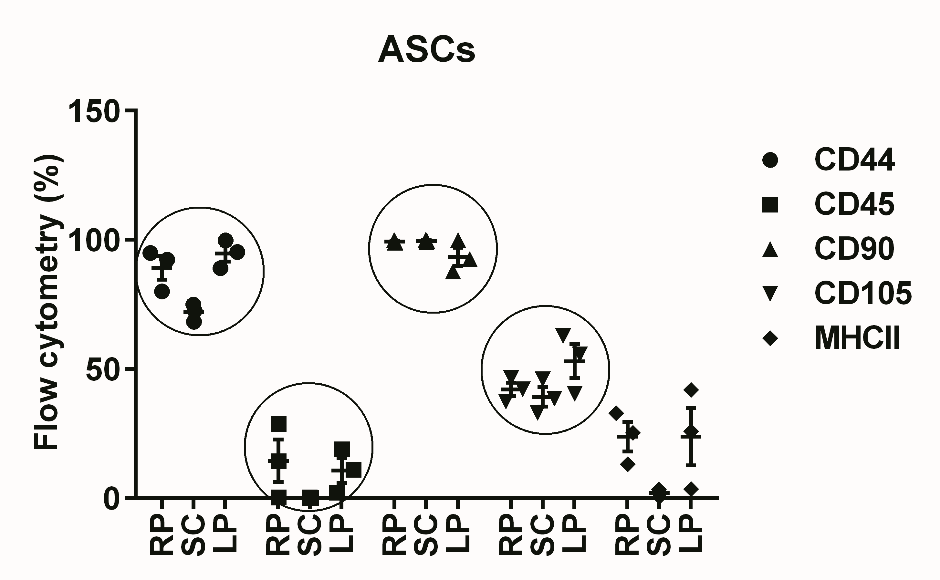


Supplementary fig.1a. Flow cytometry analysis for specific stem cells markers including CD44, CD90, CD105, CD45 and major histocompatibility complex II (MHCII) for ASCs isolated from retroperitoneal (RP), subcutaneous (SC) and lipoma (LP) as previously reported from our group (Arnhold et al. 2019): *Investigation of stemness and multipotency of equine adipose-derived mesenchymal stem cells (ASCs) from different fat sources in comparison with lipoma. In Stem cell research & therapy 10 (1), p. 309.*

Supplementary fig. 1b. **Calculation of the fluid shear stress (FSS) according to Zhou et al (2010)^1^**.

$$= \frac{3\cdot\pi\cdot\mu\cdot\theta_{max}\cdot L^{2}}{4\cdot h_{0}^{2}\cdot T}$$

= FSS (Pa)$, \mu$ = fluid viscosity (Pa s), $\theta_{max}$ = maximum rocking angle (rad), $L$ = culture dish diameter (mm), $h_{0}$ = fluid depth (mm), $T$ = rocking period, turns/minute (s)

**Fluid shear stress setup**

10°

^1^ Zhou X, Liu D, You L, Wang L. Quantifying fluid shear stress in a rocking culture dish. Journal of biomechanics 2010; 43 (8): 1598–602. The illustration is presented as previously reported *(Elashry et al. 2019):* Elashry MI, Gegnaw ST, Klymiuk MC, Wenisch S, Arnhold S. *Influence of mechanical fluid shear stress on the osteogenic differentiation protocols for Equine adipose tissue-derived mesenchymal stem cells. Acta Histochem. 2019; 121:344–53.*
